# Assortative Mating and Linkage Disequilibrium

**DOI:** 10.1534/g3.116.034967

**Published:** 2016-10-26

**Authors:** Philip W. Hedrick

**Affiliations:** School of Life Sciences, Arizona State University, Tempe, Arizona 85287

**Keywords:** heterozygosity, inbreeding, phenotype, positive-assortative mating, selective mating

## Abstract

Assortative mating has been suggested to result in an increase in heritability and additive genetic variance through an increase in linkage disequilibrium. The impact of assortative mating on linkage disequilibrium was explicitly examined for the two-locus model of Wright (1921) and two selective assortative mating models. For the Wright (1921) model, when the proportion of assortative mating was high, positive linkage disequilibrium was generated. However, when the proportion of assortative mating was similar to that found in some studies, the amount of linkage disequilibrium was quite low. In addition, the amount of linkage disequilibrium was independent of the level of recombination. For two selective assortative models, the amount of linkage disequilibrium was a function of the amount of recombination. For these models, the linkage disequilibrium generated was negative mainly because repulsion heterozygotes were favored over coupling heterozygotes. From these findings, the impact of assortative mating on linkage disequilibrium, and consequently heritability and additive genetic variance, appears to be small and model-specific.

In population and evolutionary genetics, it is generally assumed that there is random mating in a population. Major exceptions to this assumption include inbreeding (more mating between relatives than expected by chance), assortative mating (more mating between phenotypically similar individuals than expected by chance), and selective mating (in which mating success varies for different phenotypes or genotypes). To differentiate between these exceptions to random mating, Lewontin *et al.* (1968) stated that “Selective mating is character specific and involves gene frequency change; assortative mating is character specific but involves no gene frequency change; inbreeding is not character specific and involves no gene frequency change.” For assortative mating, the element of no gene frequency change implies that “all genotypes make the same average genetic contribution to the next generation” (Spencer 1992). In addition, both inbreeding and assortative mating are expected to increase the frequency of homozygotes compared to that expected from random mating.

However, this definition of assortative mating does not consider various models of selective mating in which there is also an increased probability of mating between individuals of the same phenotype. Lewontin *et al.* (1968) pointed out that both selective mating and assortative mating might occur in a given population and that, potentially, their individual effects could be separated out. Spencer (1992) showed that general examples of disassortative mating, more matings between phenotypically different individuals than expected by chance, resulted in changes in allele frequency and are therefore actually examples of selective mating using the Lewontin *et al.* (1968) definition. As a result, Spencer (1992) suggested that the “distinction between assortative and selective mating made by Lewontin *et al.* (1968) is unproductive.”

Theoretical analyses of assortative mating have concluded that it can generate linkage disequilibrium, and consequently influence estimates of heritability and additive genetic variance (Wright 1921; Crow and Felsenstein 1968; however, see Lande 1977), a finding now being applied to detailed analysis of phenotypic variation in human populations (Robinson *et al*. 2017). However, it is not clear how much linkage disequilibrium can be generated by assortative mating. Further, the effects of selective mating, in which there is also an increased probability of mating between phenotypically similar individuals, has not been examined to determine whether the conclusions for pure assortative mating are similarly true. Here, I examine whether the amount and type of linkage disequilibrium generated by assortative mating depends upon the assumptions of the model of assortative mating. In the discussion, the different models of assortative mating are discussed in an attempt to understand when, and if, they are consistent with assortative mating in natural populations.

For clarity, note that in general the terms assortative mating and positive-assortative mating are used interchangeably and the terms disassortative and negative-assortative mating are used interchangeably. The impact of assortative mating on genotypic frequencies was examined in the early days of population genetics by Jennings (1916), Wentworth and Remick (1916), and Fisher (1918). Wright (1921) subsequently examined the effect of a two-locus assortative mating model and generalized these results to multiple loci using path coefficient analysis. For the details of this elegant examination of different degrees of assortative mating for the *n* locus model, see Wright (1921) and Crow and Felsenstein (1968).

In his two-locus model, Wright (1921) assumed that the two loci were equivalent in effect, all alleles had a frequency of 1/2, and that there are five phenotypic groups in the population denoted by the number of alleles of a given type summed over the two loci. That is, the different two-locus genotypes can be classified into five phenotypic categories (Table 1) based on the number of capital *A* or *B* alleles (Wright 1921) or number of *A*_1_ or *B*_1_ alleles (Crow and Kimura 1970). Here, I have organized the genotypes of Crow and Kimura (1970) into their four constituent gametes. Notice that, for phenotypic value 2, there are four different genotypes: two of which are double heterozygotes, one with coupling gametes (*A*_0_*B*_0_ and *A*_1_*B*_1_), and one with repulsion gametes (*A*_0_*B*_1_ and *A*_1_*B*_0_).

**Table 1:** The two-locus genotypes and their phenotypic values

Genotype		
Wright (1921)	Crow and Kimura (1970)	Phenotype
*AABB*	*A*_1_*B*_1_/*A*_1_*B*_1_	4
*AABb*, *AaBB*	*A*_0_*B*_1_/*A*_1_*B*_1_, *A*_1_*B*_0_/*A*_1_*B*_1_	3
*AaBb*, *AAbb*, *aaBB*	*A*_0_*B*_1_/*A*_0_*B*_1_, *A*_1_*B*_0_/*A*_1_*B*_0_, *A*_0_*B*_0_/*A*_1_*B*_1_, *A*_0_*B*_1_/*A*_1_*B*_0_	2
*aaBb*, *Aabb*	*A*_0_*B*_0_/*A*_0_*B*_1_, *A*_0_*B*_0_/*A*_1_*B*_0_	1
*aabb*	*A*_0_*B*_0_/*A*_0_*B*_0_	0

The assortative mating model of Wright (1921) assumed that these five phenotypic classes assorted into phenotypically homogenous groups. The frequencies of these groups are the total genotypic frequencies of the phenotype that makes up this group. For example, the frequency of the phenotype 0 group is the frequency of genotype *A*_0_*B*_0_/*A*_0_*B*_0_, the frequency of the phenotype 1 group is the sum of the frequency of genotypes *A*_0_*B*_0_/*A*_0_*B*_1_ and *A*_0_*B*_0_/*A*_1_*B*_0_, and so on. This model consequently results in an equal contribution for each genotype to the next generation and consequently, no change in allele frequency.

These groups are then assumed to be isolated from each other and, subsequently, there is random mating within each group so that each phenotypic group separately produces gametes and genotypes for the next generation. In this model, there is one type of mating within both the phenotypic 0 and 4 groups, three different types of matings within both the phenotypic 1 and 3 groups, and 10 different types of matings within the phenotypic 2 group.

Analysis of this model for unlinked loci, where all alleles had a frequency of 1/2, resulted in the conclusion that the two extreme types, *AABB* and *aabb* (Wright 1921) or *A*_1_*B*_1_/*A*_1_*B*_1_ and *A*_0_*B*_0_/*A*_0_*B*_0_ (Crow and Kimura 1970), would increase in frequency because they “can produce progeny only like themselves. Therefore, the occurrence of an extreme type is an irreversible process: or, in a different vocabulary … represent absorbing barriers” (Crow and Kimura 1970). In other words, the conclusion was that assortative mating resulted in both an increase in homozygosity and an increase in gamete frequencies containing like alleles (positive linkage disequilibrium) at the two loci, *AB* or *ab* (Wright 1921) or *A*_1_*B*_1_ and *A*_0_*B*_0_ (Crow and Kimura 1970).

## Methods

### Assortative mating

Below, I will examine the two-locus assortative mating model of Wright (1921) and include partial assortative mating, that is, a proportion *A* of the population mates assortatively and a proportion (1 – *A*) of the population mates at random. Wright (1921) briefly examined some cases of partial assortative mating using *m* as the correlation between mates. From numerical examination, it appears that *A* = *m* because the correlation between mates is equal to *A* and the same equilibrium results occur here as in the examples given by Wright (1921). It is unlikely that populations would have only assortative mating, making important the examination of the impact of partial assortative mating on linkage disequilibrium.

In addition, Wright (1921) briefly examined disassortative (negative-assortative) mating for two loci. As for the assortative mating model, mating groups were isolated and then assumed to randomly mate within these groups. For his disassortative mating model, there were three groups, the matings between the individuals that had phenotypic values of 0 and 4, the matings between the individuals that had phenotypic values of 1 and 3, and the matings between the individuals that had phenotypic values of 2 (Table 2). Given that there were these three classes of matings that differed in phenotypic value by 4, 2, and 0, the phenotypic correlation between mates was – 1. Again, we can assume that there is partial disassortative mating with a proportion *A* of disassortative mating and a proportion (1 – *A*) of random mating.

**Table 2:** The two-locus matings between different phenotypes when there is disassortative mating

Mating	Wright (1921)	Crow and Kimura (1970)
0 × 4	*aabb* × *AABB*	*A*_0_*B*_0_/*A*_0_*B*_0_ × *A*_1_*B*_1_/*A*_1_*B*_1_
1 × 3	*Aabb* or *aaBb* × *AaBB* or *AABb*	*A*_0_*B*_0_/*A*_0_*B*_1_ or *A*_0_*B*_0_/*A*_1_*B*_0_ × *A*_0_*B*_1_/*A*_1_*B*_1_ or *A*_1_*B*_0_/*A*_1_*B*_1_
2 × 2	*aaBB*, *AAbb*, *AaBb*	*A*_0_*B*_1_/*A*_0_*B*_1_, *A*_1_*B*_0_/*A*_1_*B*_0_, *A*_0_*B*_1_/*A*_1_*B*_0_, *A*_0_*B*_0_/*A*_1_*B*_1_

These matings include reciprocal matings for the 0 × 4 and 1 × 3 matings and random mating between the genotypes for the 2 × 2 matings.

### Selective assortative mating model I

Assortative mating that also includes selection can be modeled by examining the 100 different mating types between the 10 two-locus genotypes (or 55 different matings if reciprocal matings are assumed equal). Using the phenotypic values in Table 1, these mating can be considered matings between the same phenotype (assortative matings) or not. Overall, there are 18 different matings between the same phenotype if reciprocal matings are assumed equal and gamete constitution is included, one for each of the phenotypic 0 and 4 classes, three for each of the phenotypic 1 and 3 classes, and 10 for the phenotypic 2 class. For example, for phenotypic 1 class, in which both mates have a phenotypic value of 1 using the notation of Crow and Kimura (1970), there are three different assortative matings; *A*_0_*B*_0_/*A*_0_*B*_1_ × *A*_0_*B*_0_/*A*_0_*B*_1_, *A*_0_*B*_0_/*A*_0_*B*_1_ × *A*_0_*B*_0_/*A*_1_*B*_0_, and *A*_0_*B*_0_/*A*_1_*B*_0_ × *A*_0_*B*_0_/*A*_1_*B*_0_.

A complete assortative mating model, in this case, can be modeled by determining the expected frequency of all mating types and then assuming that only those matings between like types are successful matings. Matings between different phenotypes could be unsuccessful because of subsequent mating incompatibility or an inability to reproduce due to sterility or inviability. In this case, the total proportion of the 18 assortative matings can be used to standardize the proportions of these assortative matings.

In addition, this model can be modified for partial assortative mating where matings that are at random are included as well. To do this, first the frequencies of the 18 assortative matings are standardized to sum to 1 as above. Then, the overall frequencies of the different matings can be calculated as

$$M_{ij}=AM_{ij}^{A}+(1-A)M_{ij}^{1-A}$$ (1)

where *A* and 1 – *A* are the proportions of assortative and random mating, $M_{ij}^{A}$ is the standardized frequency of assortative mating between genotype *i* and genotype *j*, and $M_{ij}^{1-A}$is the frequency of random mating between genotype *i* and genotype *j*. From numerical examination, *A* in this model is equal to the correlation between mates, as in the Wright (1921) model.

### Selective assortative mating model II

Assortative mating that includes selection can also be modeled in the following approach, in which there are different levels of phenotypic similarity and mating propensity between mates. To do this, the matings can be first ranked by the similarity of the two mates and these values can then be used to weight their relative levels of assortative mating success. For example, the similarity of the mating pairs in their phenotypic score is shown in Table 3 for five matings, where the first mate is *A*_0_*B*_0_/*A*_0_*B*_0_ and the second mate differs by 0 to 4 alleles. The difference in phenotypic values between mates can be additively scaled to provide a relative mating level as

**Table 3:** The phenotypic difference (*d*) between mates for the selective assortative mating model when the first mate is *A*_0_*B*_0_/*A*_0_*B*_0_ and the second mate varies

Mating	Phenotypic difference (*d*)	Mating value (*m*)
*A*_0_*B*_0_/*A*_0_*B*_0_ × *A*_0_*B*_0_/*A*_0_*B*_0_	0	1
*A*_0_*B*_0_/*A*_0_*B*_0_ × *A*_0_*B*_0_/*A*_0_*B*_1_	1	1 – *s*/4
*A*_0_*B*_0_/*A*_0_*B*_0_ × *A*_0_*B*_0_/*A*_1_*B*_1_	2	1 – *s*/2
*A*_0_*B*_0_/*A*_0_*B*_0_ × *A*_0_*B*_1_/*A*_1_*B*_1_	3	1 – 3*s*/4
*A*_0_*B*_0_/*A*_0_*B*_0_ × *A*_1_*B*_1_/*A*_1_*B*_1_	4	1 – *s*

The relative mating values for assortative mating can be calculated from 1 – *sd*/4.

$$m=1-\frac{sd}{4}$$ (2a)

where *d* is the difference in phenotypic values between mates. Here, *s* is the difference between matings that have the same phenotypic value and matings that have the greatest difference, the highest *d* value. When *s* = 1, *m* has a range from 0 to 1. Each mating frequency is then weighted by these relative mating levels and standardized to sum to unity as above.

Disassortative mating can be examined for this model in a similar manner using the following expression

$$m_{D}=1-\frac{s(4-d)}{4}$$ (2b)

where *d* and *s* are defined as above. In this case, when *s* = 1, *m_D_* has a value of 0 for matings between individuals with the same phenotypic value and 1 for matings that are the most phenotypically divergent possible (*d* = 4).

Note that these two selective mating models are related in that selective assortative model I assumes only one type of assortative mating is successful, those between individuals with the same phenotype. In other words, model I assumes that all matings in Table 3, except the one with no phenotypic difference, have mating values of 0. However, these two models are usefully parameterized in different ways; model I uses a partial assortative mating approach like the Wright (1921) model while model II allows differential values for matings that have different levels of phenotypic differences.

### Generation of gametes and progeny

Once the proportions of different matings have been determined for these models, then the proportions of genotypes for the next generation are determined. For each genotype in a mating, the proportions of gametes and then the genotypic proportions in the progeny are determined. Table 4 gives an example for the mating *A*_0_*B*_0_/*A*_0_*B*_1_ × *A*_0_*B*_0_/*A*_1_*B*_1_, where *c* is the proportion of recombination between the loci. For example, the proportion of progeny with genotype *A*_0_*B*_0_/*A*_0_*B*_0_ from this mating is (1 – *c*)/4.

**Table 4:** The gamete and progeny frequencies for the mating type *A*_0_*B*_0_/*A*_0_*B*_1_ × *A*_0_*B*_0_/*A*_1_*B*_1_

		*A*_0_*B*_0_/*A*_0_*B*_1_			
		*A*_0_*B*_0_ (½)	*A*_0_*B*_1_ (½)	*A*_1_*B*_0_ (0)	*A*_1_*B*_1_ (0)
	*A*_0_*B*_0_ [(1 – *c*)/2]	(1 – *c*)/4	(1 – *c*)/4	0	0
*A*_0_*B*_0_/*A*_1_*B*_1_	*A*_0_*B*_1_ (*c*/2)	*c*/4	*c*/4	0	0
	*A*_1_*B*_0_ (*c*/2)	*c*/4	*c*/4	0	0
	*A*_1_*B*_1_ [(1 – *c*)/2]	(1 – *c*)/4	(1 – *c*)/4	0	0

### Measures of linkage disequilibrium and fixation

There are multiple measures of linkage disequilibrium that have been used. First, the level of linkage disequilibrium can be measured by

$$D=x_{00}-p_{0}q_{0}$$ (3a)

which is the difference between the observed frequency of gamete *A*_0_*B*_0_ (*x*_00_) and the expected frequency (*p*_0_*q*_0_), assuming random association of the alleles at different loci, where *p*_0_ and *q*_0_ are the frequencies of alleles *A*_0_ and *B*_0_ (Lewontin and Kojima 1960). *D* can also be calculated from

$$D=x_{00}x_{11}-x_{01}x_{10}$$ (3b)

where *x*_00_ and *x*_11_ are the frequencies of the coupling gametes *A*_0_*B*_0_ and *A*_1_*B*_1_, and *x*_01_ and *x*_10_ are the frequencies of the repulsion gametes *A*_0_*B*_1_ and *A*_1_*B*_0_.

A widely-used measure of linkage disequilibrium is the square of the correlation coefficient or

$$r^{2}=\frac{D^{2}}{p_{0}p_{1}q_{0}q_{1}}$$ (3c)

where *p*_1_ and *q*_1_ are the frequencies of alleles *A*_1_ and *B*_1_ (Hill and Robertson 1968). When the allele frequencies are the same at the two loci, this measure has a range from 0 to 1. The upper limit is lower when the allele frequencies are different at the two loci.

The effect of assortative mating on heterozygosity can be measured by the fixation index (an estimate of inbreeding)

$$F=\frac{H_{E}-H_{O}}{H_{E}}$$ (4a)

where *H_E_* and *H_O_* are the expected heterozygosity with Hardy–Weinberg proportions and the observed heterozygosity, respectively. The range of this measure is from 0 when the population is in Hardy–Weinberg proportions to 1 when the observed heterozygosity is 0. When the observed heterozygosity is higher than the expected heterozygosity, this measure becomes negative.

In a model with both inbreeding and random mating, the equilibrium genotypic frequencies are not simple extensions of the single-locus equilibria (Bennett and Binet 1956; Weir and Cockerham 1973). In fact, there is an excess of both double heterozygotes and of double homozygotes and a deficiency of single homozygotes when compared to single-locus inbreeding equilibria values. Bennet and Binet (1956) showed that this effect could be measured by

$$d_{H}=H_{O(AB)}-H_{O(A)}H_{O(B)}$$ (4b)

where *H_O_*_(_*_AB_*_)_ is the observed frequency of genotypes heterozygous at both loci and *H_O_*_(_*_A_*_)_ and *H_O_*_(_*_B_*_)_ are the observed heterozygosities at loci *A* and *B*, respectively.

### Data availability

The author states that all data necessary for confirming the conclusions presented in the article can be generated using the approaches described fully within the article. Computer programs in Fortran are available from the author for researchers who want to determine these measures for various parameter values.

## Results

### Assortative mating

**Figure 1:**
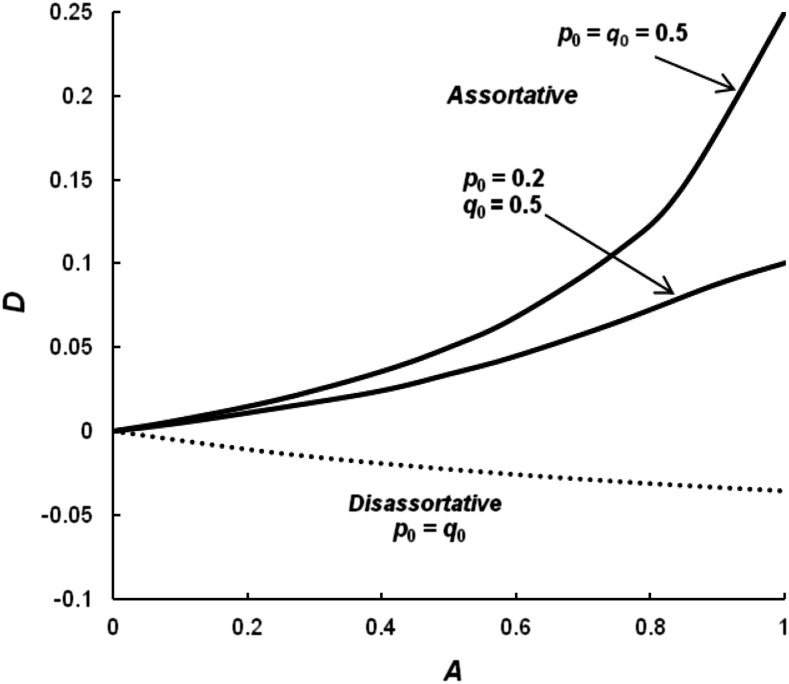
The level of linkage disequilibrium generated by the assortative mating model of Wright (1921) as measured by *D* for assortative mating with *p*_0_ = *q*_0_ or *p*_0_ = 0.2 and *q*_0_ = 0.5 (solid lines) or disassortative mating with *p*_0_ = *q*_0_ (dotted line).

Figure 1 gives the level of linkage disequilibrium at equilibrium as measured by *D* generated by assortative mating using the Wright (1921) model for different proportions of assortative mating for two different combinations of allele frequencies. First, when the allele frequencies at the two loci are both 1/2 and *A* = 1, then *D* = 0.25, its maximum value. When the allele frequencies are equal, the frequencies of the coupling gametes *A*_0_*B*_0_ and *A*_1_*B*_1_ are *p*_0_ = *q*_0_ and *p*_1_ = *q*_1_, respectively, and the frequencies of the repulsion gametes are 0. However, for smaller values of *A*, *D* is quite low. For example, when *A* = 0.25, *D* is only 0.0192. In other words, the conclusion of Crow and Kimura (1970) about the irreversible accumulation of extreme types from assortative mating only occurs when there is complete assortative mating. When the two loci differ in allele frequency, then the magnitude of *D* is less. As an example, the equilibrium level when *p*_0_ = 0.2 and *q*_0_ = 0.5 is given in Figure 1. In this case, when *A* = 1 the maximum *D* is 0.1, and when *A* = 0.25, *D* is only 0.0140.

As expected, the allele frequency does not change and the frequency of heterozygotes is reduced. When the allele frequencies are equal at the two loci, then the estimate of inbreeding is

$$F=\frac{A}{4-3A}$$ (5a)

as shown by Wright (1921) where *A* = *m* in his notation. Interestingly, this is equivalent to the expectation for partial-full sib mating (Hedrick and Cockerham 1986) and lower than that expected for partial selfing if *S* = *A* where at equilibrium,

$$F=\frac{S}{2-S}$$ (5b)

where *S* is the proportion of selfing. The reason for the difference from partial selfing is that there are matings between individuals with the same phenotype but not the same genotype. The pattern of *d_H_* is similar to that seen for partial selfing (Hedrick 1990) in that the maximum value of 0.068 is for a high level of *A* (0.82) when *p*_0_ = *q*_0_ = 0.5 and declines to 0 as *A* approaches either 0 or 1.

When there is disassortative mating, linkage disequilibrium is negative and even smaller absolutely than that generated by assortative mating. When *A* = 1, the level reaches a minimum *D* of −0.0357, and when *A* = 0.25, *D* is only −0.0132. The linkage disequilibrium generated by disassortative mating is negative because there are more repulsion gametes than expected by chance. The small effect on linkage disequilibrium appears to occur because, even with *A* = 1, only 2.9% of the matings are between individuals with phenotypes 0 and 4, while 45.7% are between individuals with phenotypes of 1 and 3, and even more, 51.4%, are between individuals with phenotypic values of 2. For this model, there is a change in allele frequency, so disassortative mating in this case is actually a case of selective mating. In particular, when both loci have the same initial frequency and *A* = 1, the alleles change to a frequency of 1/2 and remain there, indicating a stable polymorphism.

**Figure 2:**
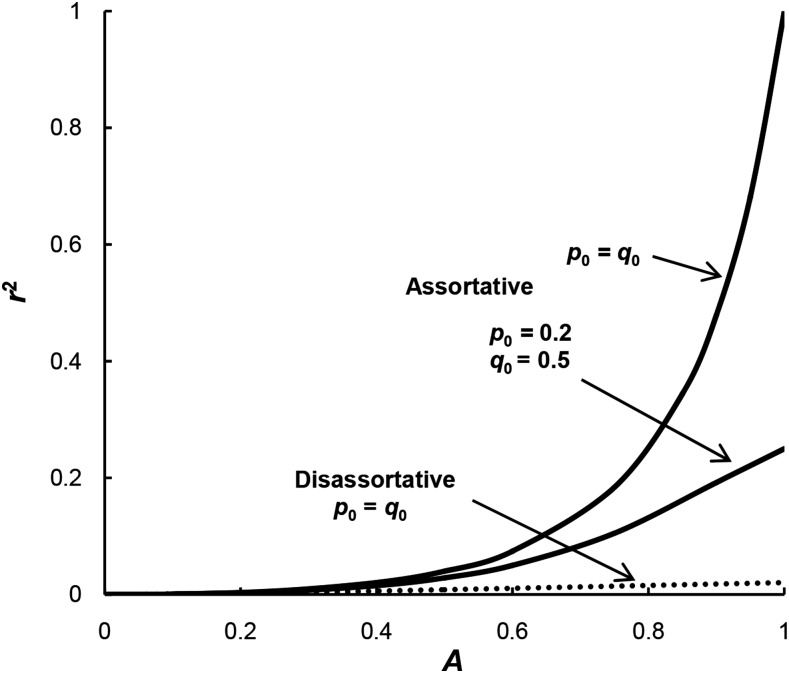
The level of linkage disequilibrium generated by the assortative mating model of Wright (1921) as measured by *r*^2^ for assortative mating with *p*_0_ = *q*_0_ or *p*_0_ = 0.2 and *q*_0_ = 0.5 (solid lines) or disassortative mating with *p*_0_ = *q*_0_ (dotted line).

For assortative mating and equal allele frequencies at the two loci, then *r*^2^ = 1 when *A* = 1 (Figure 2). However, as *A* declines then *r*^2^ quickly drops and when *A* = 0.25, *r*^2^ is only 0.0059. When the two loci differ in allele frequency, then the magnitude of *r*^2^ is even less. For example, the equilibrium levels when *p*_0_ = 0.2 and *q*_0_ = 0.5 are given in Figure 2. In this case, when *A* = 1, the maximum *r*^2^ is 0.25 and when *A* = 0.25, *r*^2^ is only 0.0047. When there is disassortative mating, linkage disequilibrium using *r*^2^ is also small. When *A* = 1, the level reaches a maximum *r*^2^ of only 0.0204 and when *A* = 0.25, *r*^2^ is only 0.0028.

For assortative mating when *A* = 1, Ghai (1973) found the equilibrium frequencies of the four gametes for different frequencies. Specifically, Ghai (1973) found that the equilibrium frequencies of gametes *A*_0_*B*_0_, *A*_0_*B*_1_, *A*_1_*B*_0_, and *A*_1_*B*_1_ are *p*_0_, 0, *q*_0_ – *p*_0_, and *q*_1_, respectively, when *p*_0_ < *q*_0_, *p*_0_, 0, 0, and *p*_1_ when *p*_0_ = *q*_0_, and *q*_0_, *p*_0_ – *q*_0_, 0, and *p*_1_, respectively, when *p*_0_ > *q*_0_ (Table 5). With these gametic frequencies when *A* = 1, the equilibrium values of *D* and *r*^2^ can be calculated. For example, when *p*_0_ < *q*_0_, *D* = *p*_0_*q*_1_, and *r*^2^ = *p*_0_*q*_1_/*p*_1_*q*_0_. When *p*_0_ = 0.2 and *q*_0_ = 0.5, then *A*_0_*B*_0_ = 0.2, *A*_0_*B*_1_ = 0, *A*_1_*B*_0_ = 0.3, and *A*_1_*B*_1_ = 0.5; *D* = 0.1 and *r*^2^ = 0.25.

**Table 5:** The initial gamete frequencies and the equilibrium genotype frequencies and linkage disequilibrium for complete assortative mating (*A* = 1) and maximum inbreeding (Ghai 1973)

			Assortative mating			
Gamete	Initial frequency	Genotype	*p*_0_ < *q*_0_	*p*_0_ = *q*_0_	*p*_0_ > *q*_0_	Inbreeding
*A*_0_*B*_0_	*p*_0_*q*_0_	*A*_0_*B*_0_/*A*_0_*B*_0_	*p*_0_	*p*_0_	*q*_0_	*p*_0_*q*_0_
*A*_0_*B*_1_	*p*_0_*q*_1_	*A*_0_*B*_1_/*A*_0_*B*_1_	0	0	*p*_0_ – *q*_0_	*p*_0_*q*_1_
*A*_1_*B*_0_	*p*_1_*q*_0_	*A*_1_*B*_0_/*A*_1_*B*_0_	*q*_0_ – *p*_0_	0	0	*p*_1_*q*_0_
*A*_1_*B*_1_	*p*_1_*q*_1_	*A*_1_*B*_1_/*A*_1_*B*_1_	*q*_1_	*p*_1_	*p*_1_	*p*_1_*q*_1_
*D*	0		*p*_0_*q*_1_	*p*_0_*p*_1_	*q*_0_ *p*_1_	0
*r*^2^	0		*p*_0_*q*_1_/*p*_1_*q*_0_	1	*p*_1_*q*_0_/*p*_0_*q*_1_	0

A major aspect of this assortative mating model is that the equilibrium linkage disequilibrium is not influenced by the level of recombination (see Ghai 1973 for *A* = 1). This suggests that loci that are not linked can show the same level of linkage disequilibrium as those that are linked. However, even though the equilibrium level of linkage disequilibrium is not influenced by recombination, the approach to the equilibrium level of linkage disequilibrium is slowed by lower recombination (not shown).

### Selective assortative mating model I

**Figure 3:**
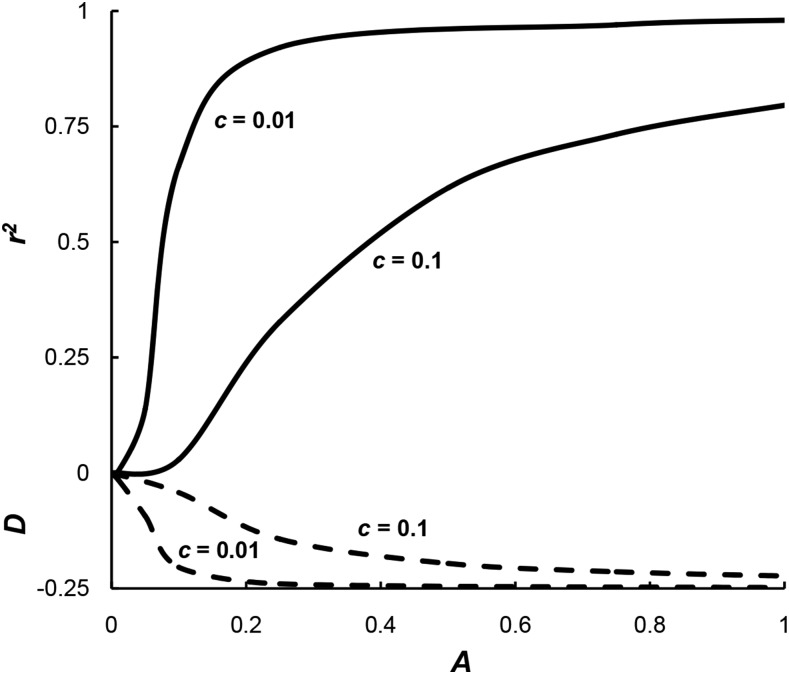
The level of linkage disequilibrium generated by selective assortative mating model I for different levels of assortative mating *A* and for two levels of recombination, *c* = 0.1 and *c* = 0.01, as measured by *D* (broken lines and < 0) or *r*^2^ (solid lines and > 0).

The results from this selective assortative mating model are quite different from the assortative mating model of Wright (1921). First, the level of linkage disequilibrium at equilibrium is highly dependent upon the rate of recombination. To illustrate, Figure 3 gives the linkage disequilibrium for *c* = 0.01 and *c* = 0.1 for both *D* (broken lines) and *r*^2^ (solid lines) for different levels of assortative mating when initially all alleles have a frequency of 1/2 and there is no linkage disequilibrium. The level of disequilibrium is significantly higher for the lower recombination rate of *c* = 0.01, reaching nearly the most extreme levels possible even when *A* = 0.2. For no linkage (*c* = 0.5) with *A* = 1, *D* is only −0.081 and *r*^2^ is only 0.105 (not shown).

Second, the sign of linkage disequilibrium for *D* is negative, the opposite of the sign for the Wright (1921) model. In other words, there is an excess of repulsion gametes rather than an excess of coupling gametes. When there is lower recombination, repulsion heterozygotes produce gametes that are more phenotypically intermediate than do coupling heterozygotes which consequently results in an advantage for repulsion gametes and their consequent genotypes compared to coupling gametes (see *Discussion*).

Third, there is allele frequency change for this selective assortative mating model, often in a complicated manner. When the initial allele frequencies are equal, if the allele frequencies are close to the equilibrium frequency of 1/2, then the frequencies change to this equilibrium, that is, there is a stable polymorphism at both loci. For example, if *c* = 0.1, *A* = 0.25, and the initial frequencies of *A*_0_ and *B*_0_ are equal and between 0.37 and 0.63 and there is no disequilibrium between the loci, then the allele frequencies change and approach the equilibrium of 1/2. If the initial frequencies are equal and < 0.37, then they both decline to 0. Or, if they are equal and > 0.63, they both increase to 1. These regions are somewhat broadened or reduced depending upon the initial presence of negative or positive linkage disequilibrium, respectively.

However, if the initial frequencies of *A*_0_ and *B*_0_ differ, then the allele with the lower frequency goes to a frequency of 0 and the allele with the higher frequency goes to a frequency of 1. This is partly explained by the unstable equilibrium at ½ that is present for a single locus with this type of assortative mating. The presence of a second linked locus with selective assortative mating further complicates the dynamics. For example, if *c* = 0.1 and *A* = 0.25 and the initial frequencies of *A*_0_ and *B*_0_ are 0.4 and 0.42, then eventually *p*_0_ = 0 (*A*_0_ will be lost) and *q*_0_ = 1 (*B*_0_ will be fixed). If the initial frequencies of *A*_0_ and *B*_0_ are unequal but both are below 0.37, then they both will go to 0.

Fourth, unlike the Wright (1921) model, the single-locus observed heterozygosity level is not greatly influenced. For example, with *c* = 0.1 and *A* = 0.25, *F* is only 0.0071. However, the deviation *d_H_* of two-locus observed heterozygosity from single-locus predictions, is often large. For example, for *c* = 0.1 and *A* = 0.25, *d_H_* = 0.0888, mainly because the frequency of repulsion heterozygotes in the population is high at 0.312 and virtually all genotypes with a phenotypic value of 2 are *A*_0_*B*_1_/*A*_1_*B*_0_ repulsion heterozygotes. The level of *d_H_* reaches a maximum at *A* =1, unlike that for the Wright (1921) model. This is in contrast to the same model for one locus in which, at the unstable equilibrium of 1/2, the equilibrium value of *F* for a given value of *A* is the same as for a given value of *S* for partial selfing in expression (5b).

### Selective assortative mating model II

**Figure 4:**
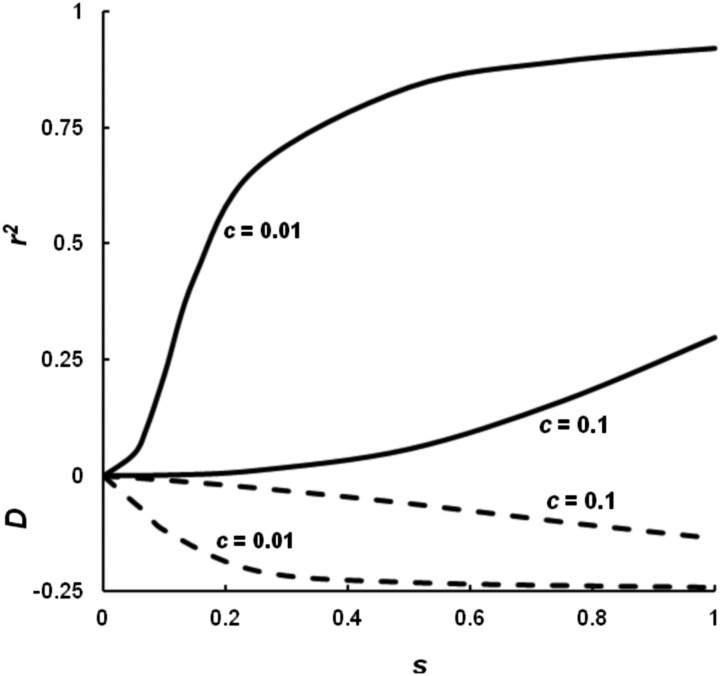
The level of linkage disequilibrium generated by selective assortative mating model II for different levels of mating selection and for two levels of recombination, *c* = 0.1 and *c* = 0.01, as measured by *D* (broken lines and < 0) or *r*^2^ (solid lines and > 0).

The results from the selective assortative mating model II are also quite different from the assortative mating model of Wright (1921) and generally similar to selective assortative mating model I, but not as extreme. Again, the levels of linkage disequilibrium at equilibrium for both *D* (broken lines) and *r*^2^ (solid lines) are highly dependent upon the rate of recombination (Figure 4). The level of disequilibrium is significantly higher for the lower recombination rate of *c* = 0.01 than for the higher level of recombination of *c* = 0.1. For no linkage (*c* = 0.5), even with *s* = 1, *D* is only − 0.015 and *r*^2^ is only 0.004 (not shown).

As for selective assortative mating model I, the sign of linkage disequilibrium for *D* is negative, the opposite of the sign for the Wright (1921) model. Also, there is allele frequency change for this selective assortative mating model, often in a complicated manner as discussed for model I. Unlike the Wright (1921) model, the single-locus observed heterozygosity level is not greatly influenced. For example, with *c* = 0.1 and *s* = 1, *F* is only 0.013 but *d_H_* is often large. For example, for *c* = 0.1 and *s* = 1, *d_H_* = 0.077, mainly because the frequency of repulsion heterozygotes in the population is high at 0.300.

Unlike both the Wright (1921) and the selective assortative mating model I where *A* is equal to the phenotypic correlation between mates, there does not appear to be such a simple relationship for this model. For example, when *s* = 1 and *c* =0.5, the correlation between mates is 0.268 (at the equilibrium with *p*_0_ = *q*_0_ = 1/2). However, at equilibrium when *s* = 1 and tight linkage with *c* = 0.01, the correlation between mates is only 0.014. This occurs because virtually all of the genotypes present in the population have a phenotypic value of 2, the population mean. The very high negative linkage disequilibrium presents results in nearly all the genotypes being either the homozygotes *A*_0_*B*_1_/*A*_0_*B*_1_ and *A*_1_*B*_0_/*A*_1_*B*_0_ or the repulsion heterozygote *A*_0_*B*_1_/*A*_1_*B*_0_, all with phenotypic values of 2. In this situation, this assortative selective mating model becomes much less effective in causing a positive correlation between mates.

When there is disassortative mating for selective assortative mating model II, linkage disequilibrium generated between the two loci is positive. For example, when *s* = 1 and *c* = 0.01, *D* = 0.180 (*r*^2^ = 0.520). For disassortative mating in this model, the mating between the two homozygotes, *A*_0_*B*_0_/*A*_0_*B*_0_ and *A*_1_*B*_1_/*A*_1_*B*_1_, has the largest difference and consequently the highest relative mating level. As a result, the two gametes *A*_0_*B*_0_ and *A*_1_*B*_1_ are favored and result in positive linkage disequilibrium. The level of linkage disequilibrium for disassortative is highest for high *s* and low recombination, as it was for assortative mating with this model.

## Discussion

There is a general impression that assortative mating generates positive linkage disequilibrium and that this can result in an increase in estimates of heritability and additive genetic variance. The conclusion here of the analysis of the two-locus model of Wright (1921) is that only when the level of assortative mating is quite high is there significant positive linkage disequilibrium generated by this assortative mating model. A caveat is that the amount of linkage disequilibrium generated is independent of the rate of recombination between the loci, suggesting that when there is enough assortative mating to have an impact on linkage disequilibrium it could also have an impact on loosely or unlinked loci. In addition, the theories of Wright (1921), Crow and Felsenstein (1968), and others predict that, if there are many loci contributing to assortative mating with this model, the impact on heritability and additive genetic variance is significant, presumably because of the cumulative effect of linkage disequilibrium between these many loci.

In their review of 1116 published empirical measurements of assortative mating, Jiang *et al.* (2013) found that the mean correlation between mates was positive at a level of 0.28. In the examination of human spousal partners, Robinson *et al.* (2016) found that the phenotypic correlation for height was 0.21 and that for body mass index was 0.25. For a different sample, Tenesa *et al.* (2016) found that the phenotypic correlation for height in human couples was 0.26. Even for these reports of significant assortative mating, the level of correlation was about the same as the example we gave above for *A* = 0.25 [*A* is both the proportion of assortative mating and equals the phenotypic correlation between mates for the Wright (1921) model and the selective assortative mating model I here] where the linkage disequilibrium generated from the Wright (1921) model using *r*^2^ was only 0.0059. In other words, it appears unlikely that even for these reported levels of assortative mating, this model of assortative mating would generate substantial linkage disequilibrium.

On the other hand, given low enough recombination, both models of selective assortative mating generate linkage disequilibrium, although it is negative between the two loci influencing the phenotype. As a result, this negative linkage disequilibrium at closely linked loci would appear not to increase estimates of heritability and additive genetic variance, but lower them. Further, the selective assortative mating models also often result in fixation, depending upon the initial frequencies, with the result that there is no polymorphism and subsequently no linkage disequilibrium at equilibrium. Overall, positive linkage disequilibrium from assortative mating, that which increases estimates of heritability and additive genetic variance, is specific to the model of assortative mating and does occur in either of the selective assortative mating models considered here.

For the Wright (1921) assortative model when there is only assortative mating (*A* = 1), when the frequency of *A*_0_ and *B*_0_ are the same (*p*_0_ = *q*_0_), the equilibrium linkage disequilibrium using *r*^2^ is 1, the maximum (Table 5). Unlike the Wright (1921) assortative mating model, inbreeding does not generate linkage disequilibrium (Weir and Cockerham 1973). To illustrate, assume that initially there are Hardy–Weinberg proportions for the two-locus genotypes and no linkage disequilibrium with initial gametic frequencies of *p*_0_*q*_0_, *p*_0_*q*_1_, *p*_1_*q*_0_, and *p*_1_*q*_1_ for gametes *A*_0_*B*_0_, *A*_0_*B*_1_, *A*_1_*B*_0_, and *A*_1_*B*_1_, respectively (Table 5). If there is only inbreeding and no random mating, then eventually there will be only the four genotypes that are homozygous for these gametes, *A*_0_*B*_0_/*A*_0_*B*_0_, *A*_0_*B*_1_/*A*_0_*B*_1_, *A*_1_*B*_0_/*A*_1_*B*_0_, and *A*_1_*B*_1_/*A*_1_*B*_1_ in the frequencies *p*_0_*q*_0_, *p*_0_*q*_1_, *p*_1_*q*_0_, and *p*_1_*q*_1_, and both *D* and *r*^2^ are equal to 0. In other words, there is no linkage disequilibrium generated when there is exclusively inbreeding.

We examined two different selective assortative mating models here, one of which considered only two categories of matings: those with the same phenotype or not (similar to Wright’s categorization) and the other matings ranked by the phenotypic similarity of the mates. For both selective assortative mating models, more linkage disequilibrium was generated for closely linked loci or the level of assortative mating was higher. The selective assortative mating models did result in changes in allele frequency, resulting in an equilibrium with equal allele frequencies when the initial allele frequencies were equal and close to 1/2. However, for lower assortative mating, looser linkage, and unequal initial allele frequencies, this equilibrium was not present and for a number of initial parameter combinations, allele frequencies went to either 0 or 1.

Interestingly, the sign of linkage disequilibrium was negative for the selective assortative models while that generated by the Wright (1921) model was positive. In contrast, for disassortative mating, the selective assortative mating model I generated positive linkage disequilibrium while the Wright (1921) model generated negative linkage disequilibrium. For the Wright (1921) model, there is an absorbing barrier for extreme phenotypes (Crow and Kimura 1970) and, in addition, the number of these individuals grows because other matings also produce these individuals and they are added to this group. The Wright model assumes that the frequencies of the assortative mating types are the frequencies of phenotypes (sum of genotypes with that phenotype). For example, in Wright (1921), the frequency of *A*_1_*B*_1_/*A*_1_*B*_1_ (and the frequency of mating type *A*_1_*B*_1_/*A*_1_*B*_1_ × *A*_1_*B*_1_/*A*_1_*B*_1_) is 1/16.

The selective mating models here assume either that mating occurs at random and then that only those that are assortative stay together or are successful (selective assortative mating model I), or that there is random mating and then there is differential success based on how similar the mates are (selective assortative mating model II). For example, in both of these, the initial frequency of mating *A*_1_*B*_1_/*A*_1_*B*_1_ × *A*_1_*B*_1_/*A*_1_*B*_1_ is 1/256. This is then standardized but is still much less frequent than for the same mating in the Wright (1921) model. In addition, progeny from these individuals in the next generation often mate with other genotypes or phenotypes because they are uncommon, so this does not represent an absorbing barrier. If they are randomly paired with an individual with a different phenotype, say phenotype 2 (the most common), then they are selected against.

For the selective assortative mating models, when there is lower recombination, repulsion heterozygotes produce gametes that are phenotypically intermediate, mostly *A*_0_*B*_1_ and *A*_1_*B*_0_ both with phenotypic values of 1, than do coupling heterozygotes which produce mostly gametes *A*_0_*B*_0_ and *A*_1_*B*_1_ with phenotypic values of 0 and 2. The intermediate value for repulsion gametes results in a selective advantage in their consequent genotypes compared to coupling gametes because of the higher success of intermediate phenotypes being in an assortative mating with individuals of the same phenotype.

For the selective assortative mating models, matings between repulsion heterozygotes *A*_0_*B*_1_/*A*_1_*B*_0_ (phenotype of 2) with no recombination produce only progeny with phenotype 2 (1/4 *A*_0_*B*_1_/*A*_0_*B*_1_, 1/2 *A*_0_*B*_1_/*A*_1_*B*_0_, and 1/4 *A*_1_*B*_0_/*A*_1_*B*_0_). Matings between coupling heterozygotes *A*_0_*B*_0_/*A*_1_*B*_1_ produce half their progeny with phenotype 2 (*A*_0_*B*_0_/*A*_1_*B*_1_), 1/4 with phenotype 0 (*A*_0_*B*_0_/*A*_0_*B*_0_), and 1/4 with phenotype 4 (*A*_1_*B*_1_/*A*_1_*B*_1_). This gives a significant advantage to matings between repulsion heterozygotes because all their progeny have the same, and most common, phenotype. As a result, there are virtually all repulsion heterozygotes and no coupling heterozygotes at equilibrium (when there is one) for low recombination.

The Wright (1921) assortative model assumes that the different phenotypic classes assorted into phenotypically homogenous groups. These groups are then assumed to be isolated from each other and subsequently randomly mate within each group so that each phenotypic group separately produces gametes and genotypes for the next generation in the frequency of the phenotype that makes up the group. This model results in an equal contribution for each genotype to the next generation and consequently no change in allele frequency. On the other hand, the selective assortative mating model I above assumes that, initially, pairing occurs randomly among the different phenotypes. Subsequently, matings between different phenotypes are unsuccessful because of subsequent mating incompatibility or inability to reproduce due to sterility or inviability.

The selective assortative mating model II provides a different reflection and perhaps a more realistic one of assortative mating than these models, that is, the relative value of mating success depends upon the level of phenotypic similarity of potential mates. In other words, it does not assume isolation of different phenotypic groups like the Wright (1921) assortative mating model, and does not assume overall initial random mating and subsequent higher success of assortative matings as the selective assortative mating model I. Therefore, the conclusions from selective assortative mating model II, negative linkage disequilibrium, linkage disequilibrium level as a function of the amount of recombination, changes in allele frequency often resulting in loss of polymorphism, low amounts of loss of single-locus heterozygosity, and higher amounts of two-locus deviation, appear to be reasonable expectations that would be observed from assortative mating in natural populations.

Jiang *et al.* (2013) provided an important perspective on the factors involved in assortative mating. First, they suggested that assortative mating could evolve because there is direct or indirect selection on phenotypic mate similarity. That is, selection could act directly, favoring either phenotypically similar mates, or selection could act indirectly, influencing the fitness of offspring of the mating pair based on the phenotypic similarity of the parents. Second, assortative mating could be “an incidental consequence of temporal, mechanical, and physiological constraints” (Jiang *et al.* 2013). For example, temporal segregation of mates that is associated with some phenotype could result in assortative mating.

In addition, the extent and manner of assortative mating depends upon the level of mate choice possible (for a general introduction, see Shuster and Wade 2003). For example, if mating occurs with one of the first mates encountered, it is unlikely to be very selective, while if many potential mates are encountered before mating, it is likely to more selective. The numbers of potential mates encountered before mating depends upon the species, the population density, receptivity of the mates, and other factors. Similarly, if mate choice results from only female choice, then it might be quite different than if both female choice and male–male interaction determined the success of a mating. It is possible that the extent of assortative mating could be higher when female mate choice is stronger. For an introduction to the factors involved in human mate choice, see Geary *et al.* (2004).

Given this perspective, can we suggest which of the assortative models examined here represent the best description of assortative mating in natural populations and, in particular, human populations? First, it is unlikely that any one assortative mating model provides a universal description of assortative mating, given the potential causes of assortative mating mentioned above and the diversity of organisms and traits exhibiting assortative mating. Second, all three assortative mating models here are based on assumptions that might apply to some populations. For example, the Wright (1921) model suggests that the population is separated into different phenotypic groups before random mating within these groups and such segregation might result from the incidental factors suggested above. The selective assortative mating model I suggests that the only successful matings are between phenotypically identical individuals and this might occur because there is direct or indirect selection on mate similarity as suggested above. The selective assortative mating model II here provides a more general approach to assortative mating with a graduated preference given to more similar mates and is probably the closest of the models to assortative mating in humans. Research into both the cause and level of assortative mating and the observed type and amount of linkage disequilibrium between loci influencing particular phenotypes should provide insight into the importance of assortative mating causing linkage disequilibrium and the appropriateness of these, or other, models of assortative mating.
